# Editorial: Women in science: Occupational health and safety 2021

**DOI:** 10.3389/fpubh.2022.1064075

**Published:** 2022-10-28

**Authors:** Caterina Ledda, Martine Duclos, Begoña Martínez-Jarreta

**Affiliations:** ^1^Occupational Medicine, Department of Clinical and Experimental Medicine, University of Catania, Catania, Italy; ^2^Department of Sport Medicine and Functional Exploration, University Hospital CHU G. Montpied, INRAE, UNH, CRNH Auvergne, Clermont Auvergne University, Clermont-Ferrand, France; ^3^Consolidate Research Group “Toximol” and GSII-03-Occupational Medicine, Aragon Institute of Health Research (IIS-Aragon) and University of Zaragoza, Zaragoza, Spain

**Keywords:** woman, women, gender medicine, health promotion, workplace, total worker health

The proportion of women and men in science, technology, engineering, and mathematics (STEM) at undergraduate levels is relatively equal, however, there is a lack of representation of women in senior positions in Public Health. According to the UNESCO Institute for Statistics (UIS) data in 2016, < 30% of researchers in STEM are women (1). In the field of Occupational Health and Safety, there are many highly influential and successful women who are contributing to the field and tackling important questions (2). Yet, female scientists are still underrepresented in various aspects of academic life. Several initiatives have been recently created to increase the visibility of women in science (e.g., awards for women in STEM). However, evidence indicates that a gender bias is still present throughout many scientific disciplines.

This Research Topic has fostered the growth and better understanding of scientific research in occupational medicine by making visible the contributions on the field of some of the most prominent researcher women from different parts of the European continent.

Curtis et al. suggest that the industry's work environment can be hostile and unsupportive for women, contributing to tradeswomen's injury risk and psychological distress.

Felszeghi presents the model, which promotes multidisciplinary health service, combined with education and research as a novel element, and will outline its impact on national and international research. Related to this research, Pina et al. allows to highlight specific areas of user-professional conflict in Primary Care. Furthermore, the inclusion of intervention proposals by the professionals allows to propose starting points for the development of complete plans. The same research group (Pina et al.) provides evidence of the psychological consequences of the perception of user violence in the Primary Health Care staff. Furthermore, it is evident that the emergence of burnout syndrome in these professionals is related to exposure to verbal or non-physical violence together with low job satisfaction.

Moreover, Kaluznaj et al. demonstrate a high prevalence of painful conditions among Latvian employees; urgent attention to diagnostics, treatment, and prevention is needed to ensure the musculoskeletal health and productivity of this population with a special focus on women and young as occupational diseases (OD) were more prevalent among women. They also reported that workers aged 18–24 years had a higher prevalence of various types of pain. The consequences of a sedentary lifestyle is highlighted.

In another investigation Matisāne et al. shows that knowledge of the English language for occupational safety and health experts working in Latvia is not sufficient.

Xia et al. demonstrated that exposure to passive smoking was a risk factor for overall decreased physical and mental health status among Chinese nurses.

Finally, following the articles received, we would like to launch the message relating to improving the adoption and implementation of Total Worker Health^®^ in workplace settings, involving multidisciplinary teams and workers in research (3).

## Author contributions

All authors listed have made a substantial, direct, and intellectual contribution to the work and approved it for publication.

## Conflict of interest

The authors declare that the research was conducted in the absence of any commercial or financial relationships that could be construed as a potential conflict of interest.

## Publisher's note

All claims expressed in this article are solely those of the authors and do not necessarily represent those of their affiliated organizations, or those of the publisher, the editors and the reviewers. Any product that may be evaluated in this article, or claim that may be made by its manufacturer, is not guaranteed or endorsed by the publisher.
